# Suicide in New Zealand

**DOI:** 10.1100/tsw.2005.74

**Published:** 2005-07-18

**Authors:** Said Shahtahmasebi

**Affiliations:** Research Centre Director, The Good Life Research Centre Trust, Christchurch, New Zealand

**Keywords:** community records, health informatics, life events, New Zealand

## Abstract

This paper explores and questions some of the notions associated with suicide including mental illness. On average, about two-thirds of suicide cases do not come into contact with mental health services, therefore, we have no objective assessment of their mental status or their life events. One method of improving our objective understanding of suicide would be to use data mining techniques in order to build life event histories on all deaths due to suicide. Although such an exercise would require major funding, partial case histories became publicly available from a coroner's inquest on cases of suicide during a period of three months in Christchurch, New Zealand. The case histories were accompanied by a newspaper article reporting comments from some of the families involved. A straightforward contextual analysis of this information suggests that (i) only five cases had contact with mental health services, in two of the cases this was due to a previous suicide attempt and in the other three it was due to drug and alcohol dependency; (ii) mental illness as the cause of suicide is fixed in the public mindset, (iii) this in turn makes psychological autopsy type studies that seek information from families and friends questionable; (iv) proportionally more females attempt, but more men tend to complete suicide; and (v) not only is the mental health-suicide relationship tenuous, but suicide also appears to be a process outcome. It is hoped that this will stimulate debate and the collaboration of international experts regardless of their school of thought.

## INTRODUCTION

Within the suicide literature, despite the large volume of work, criticisms have been aired that our knowledge of suicide is based on scant scientific evidence[1,2,3]. The study design and the quality of data has been cited as being responsible for ambiguities in the evidence. For example, a study by Blair-West et al[4] suggested a population age specific rate of 3.5% for sufferers of depression as opposed to 15% in other studies. On the other hand, Khan[5], using data from the FDA, suggested that medical intervention (antidepressant prescription) may in fact be a contributory factor and not the solution to reducing suicide rates, whilst Hall et al[6], using data from antidepressant sales and prescriptions, concluded that prescribing antidepressants is a main contributing factor in reducing the suicide rate. On the other hand, trend analysis of suicide mortality suggest cyclic (e.g. see Figure 1) and seasonal effects[7]. Such effects, if not accounted for, will make any association between antidepressants and reduced mortality rate, as proposed by Hall et al[6], tenuous and questionable as quested by Shahtahmasebi[8]. However, the author is not aware of any studies in New Zealand that have taken account of the time series properties of suicide (see Figure 1). Not surprisingly, a downward blip in the time series may easily be claimed as the result of current knowledge and policies. Conversely, an upward blip in the time series may be explained as suicide being very complex and dependent upon many variables and that more research is essential.

Fig. 1 - Deaths from Suicide, New Zealand 1948-2000

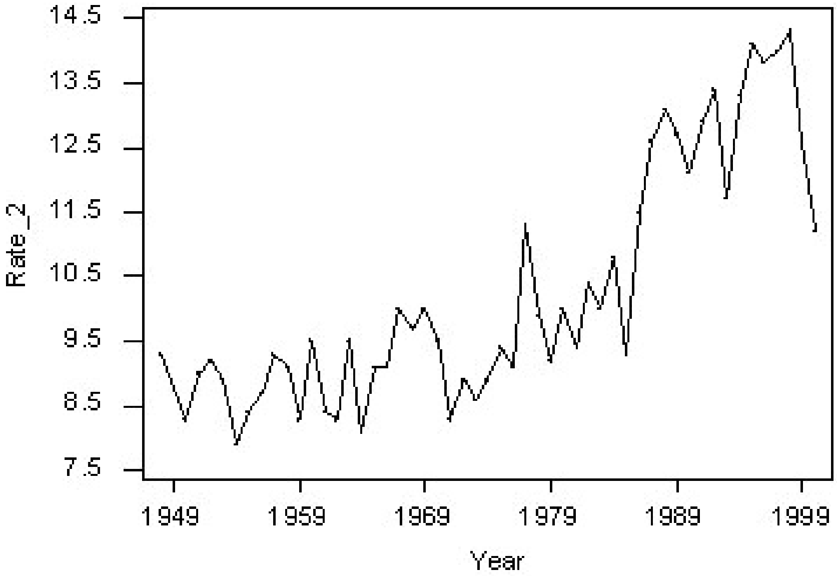

Source: (ICD-9-CM-Codes E950.0-E959.9) - NZHIS

The major problem with suicide research is that the key informants are no longer available to provide insight into the events. On the other hand, prospective designs require large samples over long periods of time, which are often ethically complex and financially prohibitive. For this reason, studies of suicide have mainly been retrospective using psychiatric records and surveys of significant others (next of kin) or have been studies of a sub-population e.g. those with failed attempted suicide, or those with a history of self-harm. Such studies are limiting as they are subjective and exclude the dynamics of life processes and may lead to spurious relationships and give undue emphasis to some variables more than others. The view that suicide is not a result of mental illness is not new[9,10,11], but whatever the context, suicide (e.g. psychiatric, medical, social) is a behavioural outcome and therefore compounded and confounded in suicide data will be high levels of measurement error and random noise due to subjectivity and unobserved/omitted characteristics associated with behavioural studies[12,13].The main issue is, therefore, the availability of objective data to gain insight into the process of suicide in order to explore and examine its causal paths. The aim of this paper is to raise relevant issues concerned with suicide, to stimulate debate and collaboration in order to develop a more coherent and holistic approach of investigating and understanding suicide.

## METHODS

One way of gathering objective life event histories is to use data mining techniques to extract data from all health and community records for all suicide cases and not just those with a psychiatric record[8]. Such an exercise requires major funding and support. Indeed, as reported[8] similar studies have been carried out in Europe and Australia. Therefore, objective data such as those presented and accepted by the coroner's inquest has been difficult to access. However, rather fortunately a sample of such data became publicly available through the media in New Zealand.Recently in an attempt to publicise the suicide issue, The Press (June 18, 2005), a New Zealand broadsheet newspaper, printed a number of recent cases that had come before the coroner. The deaths occurred in the period between September-December 2004. The newspaper reported ten very brief case histories based on evidence provided to the coroner's court together with an article based on statements and testimonies from those who had consented to talk to its reporter.

The newspaper claimed that by right it could only report the barest facts and any other details could be published only with the coroner's permission. The reported case histories were quite short, but the assumption here is that these are factual data and therefore objective. The brief case histories provided a snapshot of the employment history, health service support, history of self-harm, contact with mental health services and psychiatric diagnosis. The limitation with analysing such short life histories is that it is not possible to get a full picture of the life events and dynamics for each individual.

The availability of data through the media was fortunate as it also enabled us to explore the public's and media's perception and portrayal of suicide. This is important as the media may be the link to a feedback effect influencing the public's perception and leading to bias, i.e. causal paths of suicide by association after the event. On the other hand, this provided a unique opportunity to carry out an exploratory investigation of all suicide cases for a given period of time as opposed to only those cases with a psychiatric record.

This paper is based on the newspaper report and the case histories reported. No attempt was made to contact either the families who had spoken out or the newspaper for additional information.

## RESULTS

The Press published ten short case histories out of the 11 inquests it was covering to highlight suicide as a public health issue. There were eight males and two females with an age range of 17-93 years. The case histories were short, but provided some indication of social, educational, and employment status or problems. It is assumed that if a problem or an event was not reported in the short case histories then the event did not occur. For example, if early difficulties in life was not mentioned in the case history then the case may have had a reasonable childhood/adult life. The case histories also excluded information on the method of suicide. These topics have been dealt with elsewhere[8] and are not intended as a main feature of this short paper, nevertheless, it is important to highlight the gaps in our information.

**Social background of the sample:** apart from one student and an invalid beneficiary, the rest were a mix of professionals, self-employed, and tradesmen. At least three of the case histories mentioned life troubles/difficulties. The case history did not report any records of difficulties with the law and the police.

**Education and employment history:** the case histories did not lend themselves to describe the education history of the sample, but there were indications that apart from the student at least one person had some university training (qualification unknown). For the rest, one had not finished school, one had left school early to work on a farm, two cases had built their careers through the apprenticeship scheme. The education history of the rest was not reported in the case histories. However, the sample's employment history included one retired, one student, one on benefit, five in employment and two self-employed.

**First contact with Psychiatric Services:** four cases had contact with mental health services, two were due to drug abuse and two were due to suicide attempts. A fifth case had sought treatment for alcohol dependency.

**Morbidity background and diagnosis:** again, other than the cases who had a history of drug use, there is very little in the case histories about morbidity history. Three cases had the word 'diagnosed' mentioned in association with depression. One person was reported to have been on medication for depression, but it is not clear as to the reason and one person had no record of contact with GP or mental health services despite an apparent suicide attempt eight months earlier before his death.

**Self-harm History:** only three cases appeared to have attempted suicide before, one had no record of any contact with health services, one had briefly seen a psychiatrist and the third case had some contact with mental health services and was being monitored but does not appear to have complied with a prescribed anti-depressant.

**Prescribed Drugs:** five of the cases were receiving some sort of treatment; three had been prescribed anti-depressants, two of whom had refused to comply, and two were receiving treatment for drug dependency and were on methadone. As the method of suicide has been omitted from these case histories it is not known whether prescriptions were involved or not.

**Alcohol Abuse:** only one person had alcohol dependency for which she had sought treatment, but two cases were reported to have had blood alcohol levels of 1.5 times and 2.5 times that of the legal driving limit.

**Gender Bias and Method of Suicide:** only two of the ten cases were female suggesting men were at much higher risk of committing suicide. Indeed, the newspaper article, a subsequent editorial and letters to the editor and articles that followed in other popular magazines all led with the title "It's Okay to Talk" in big letters focusing on men. But as shown in Table 1 more women attempt suicide than men. The method of suicide was not reported in the newspaper report. As discussed previously[8] more men tend to adopt more violent methods whilst women tend to overdose. An exploratory investigation of the method of suicide suggest the possibility of switching between methods after failed attempts e.g. in their subsequent attempt women may adopt a male method such as hanging or drowning.

## COMMENTARY

Out of the ten cases only five appeared to have had some contact with the mental health services. The reason for contact with the mental health services appeared to be drug and alcohol abuse in three of the cases and a previous suicide attempt for the other two cases. One of the cases with previous suicide attempts had been prescribed anti-depressants, but apparently he had declined due to lack of time. The day before his death, an attempt had been made to monitor him through a community intensive-care team. It is interesting, though, that on the morning of his death he contacted the service and told them that he had taken a small overdose, but it was not till later that night that his body was found by other means. The other case with a previous attempt had apparently briefly seen a psychiatrist. There was no more information in the case history about a psychiatric diagnosis and paths of treatment, however, the words anxiety and drinking in the days leading to her death were mentioned in the case history.

Out of the ten case histories only four had the word depression mentioned in their case histories. In three of these case histories the word “diagnosed” in relation to depression was mentioned. One had sought treatment for alcohol dependency and was diagnosed as suffering from depression, and the other was receiving treatment for drug use and was diagnosed as exhibiting depressive symptoms. And in the third case it was mentioned that relationship problems leading to separation left the case upset and depressed and that he was diagnosed as significantly depressed. The case history does not make it clear whether the diagnosis was made after the marriage breakdown or before or after the suicide attempts. The case history for the fourth case only suggested that in the last 18 months of his life the person had been prescribed medication for depression and his health had deteriorated. He was living in a rest home, but it is not clear why and by whom he was prescribed medication (i.e. by his GP, the rest home medical staff, or a psychiatrist?). The case history does not make it clear whether the health difficulties were subsequent and due to the medication or whether he was prescribed medication in order to help him cope with the difficulties. It is interesting to note that another case who was prescribed anti-depressants had not fully complied with medication because they interfered with her creativity.

In two of the cases, blood alcohol was found to be over the legal driving limit. The case histories did not report the methods of suicide although alcohol is a depressant and an overdose of prescribed medication with alcohol is one of the more frequently used methods of committing suicide. On the other hand, the causal relationship between alcohol and depression is vague i.e. whether depressive spells are the consequences of alcohol consumption or vice versa i.e. to alleviate symptoms of depression with a few drinks.

In terms of physical and mental health outcomes there was very little known about the other five cases. Although, the inquest heard that there was a rumour that the youngest case was being picked on at school and that three months before his death he had changed his eating habits. Although no further details were provided as to how the diet had changed, there is some evidence to suggest a relationship between low cholesterol and suicide[14,15]. In all the cases the suicide had come as a shock, more so in the five cases without any health complaints and who had had no contact with a health professionals prior to death.

## DISCUSSION

There is no doubt that suicide has a profound impact on those who have lost loved ones due to suicide as is illustrated by the sub-heading to the suicide article: “When a loved one dies by suicide, it is not the end of pain, but the beginning, grieving families say.” The article is sympathetic and designed to stimulate emotions and debate to bring the discussion of suicide out in the open. The article begins with the case of one of only three cases known to have suffered from depression and how it had affected the family. The article reported that the case’s son knew his father had suffered anxiety and depression and quoted the case’s son: “I have up days and down days. It has left a big hole.” Most families spoke of guilt, anger and sorrow. “It leaves you on a roller coaster of emotions – anger, guilt, hopelessness, loss and sadness. You wonder if you could have done more to prevent this.” Indeed, some of these feelings such as loneliness, hopelessness, grief have been reported in the suicide literature [16]. Yet, out of the families who had spoken to The Press only one, a mother, mentioned that she had been trying to cope through ongoing counselling.

On the whole the article provided an impression that most victims (e.g. family and friends) of suicide were desperately looking for answer(s) to the most frequently asked question ”Why?”. Hence, it is rather difficult to understand the forceful argument by the editorial and the campaign by the newspaper for open discussion of something that the families can not themselves understand nor answer. The feelings expressed by most of the families were of guilt, hopelessness (not knowing how they could have prevented it), anger, depression, sorrow and grief. It is, therefore, quite easy for the families to (i) be influenced by *uncritical* explanations and reports in the media and (ii) become biased by association in remembering and reporting life events of the suicide case. Such bias becomes more obvious through statements such as:

> "Don't give up, especially when someone is so depressed and negative – keep on plugging …"
> "Looking back now there were little signs that I missed, but I did not see them then."
> "We had no idea he felt like this."
> "But if it does happen, there is no one person to blame."
> "I think it is a mythical idea that talking about it plants it in the minds of people. In our society there is still a stigma attached to mental health issues and suicide and people don't like to ask for help."
> "I am desperately sad we had no insight into his mental health problem and so were not able to prevent this tragedy."

It is interesting to note that both families and health professionals in their search for answers had focused on mental health even where there were no evidence of mental illness. For example, the youngest case's GP's report to the coroner had said: "I am desperately sad we had no insight into his mental health problem and so were not able to prevent this tragedy." Why should the GP automatically assume 'mental health problem' about a case reported to have been a happy and popular person with no sign of any health problems and no evidence of mental ill-health? On the other hand, it is not clear how many potential suicide cases are actually cured or prevented. What about those cases in which the mental health services had insight into their mental health problems and they still committed suicide?

It is clear that the public's perception may be an association between mental illness and uicide from the outset. More so for the relatives of the suicide cases who are looking for nswers. Therefore, a psychiatrically orientated study of suicide such as the psychological autopsy type[17,18,19,20] that relies on information gathered from families and friends of suicide cases to generate a psychiatric profile of the suicide case is biased even before it begins. The bias and measurement errors are further compounded and confounded by failure to account for the bias in methodology and in analyses. It is clear that the resulting information and profile of the cases are impressively biased and could lead to erroneous results and misleading conclusions. Therefore, it is not surprising that mental illness features very strongly as the cause of suicide.

Also set in the public's mind is the association of suicide with males from such statements by the families about the difficulties men feel in speaking out. Proportionally, more men tend to complete suicide than women, however, men tend to choose more violent methods (e.g. shooting, hanging, drowning) that increases the chance of 'success' to a complete suicide in their first attempt[8]. By the same token, data from NZHIS (see Table 1) for total public hospital discharges for suicide and self inflicted injury (E950-959) in New Zealand consistently show that proportionally more females attempt suicide and are hospitalised than men. It is unwise to attempt to shift the emphasis from one group to another, which in the longer term may lead to erosion of established links and services and a possible trend reversal. It may be more effective to address suicide as a whole.

**Table 1 –**
**Number of attempts and completed suicides by gender**

| | 1996 | | 1997 | | 1998 | |
|---|---|---|---|---|---|---|
| | Attempts | Suicide | Attempts | Suicide | Attempts | Suicide |
| Male | 2124 | 428 | 1343 | 440 | 1473 | 445 |
| Female | 1378 | 112 | 2224 | 121 | 2253 | 132 |
| Total | 3502 | 540 | 3567 | 561 | 3726 | 577 |

*Source: New Zealand Health Information Service 2000*

There was a suggestion by one of the case's relatives that we must have a plan to cope when feeling down and depressed in order to get through it. At an individual and social level a plan that may influence choice making has always been available to us, and through socio-political and other policies we may have lost our skills to utilise it. The plan is our spiritual beliefs, faith and practices. Recent research has provided some evidence that spiritual beliefs and practices influence health outcomes through an improved quality of life, quicker response to and therefore less use of medication and more effective treatment[21].

The suicide article in The Press concluded by providing details of the Ministry of Health suicide prevention material. It is very interesting to note that one of the suggestions is that if people are worried about someone they should approach a GP, community mental health service, the nearest hospital or psychiatric unit. It is also interesting to note that a draft policy document's objectives include 'improve early identification and intervention'. However much this may sound like good advice, judging by the case histories of the ten cases in which no one had suspected suicide even in the cases of those who had mental health support and the large number of risk factors associated with suicide, it would almost certainly place a very large proportion of the public under the monitoring of the mental health services.

In New Zealand, like other societies, suicide is socially unacceptable and possibly through its association with mental illness is a social stigma. The subsequent editorial (The Press, June 23, 2005) headed "Breaking the Taboo" criticised the New Zealand Coroner's Act restricting media access and reporting of suicide. The editorial attempted to justify the importance of learning to deal with suicide openly by reminding us that families of suicide cases are permanently affected by their loss and burdened by guilt and unanswered questions. It further claimed that suicide afflicts men more than women and is closely associated with depression, relationship breakdown and alcohol misuse. Given the discussion above, it is interesting to note the 'uncritical' approach of dealing with suicide both in the media and the literature.

It is time for appropriately designed good quality research to gain real insight into suicide as a behavioural process outcome. However, presently, it is wise to take a step back and take a self-critical look at our knowledge and understanding of suicide, and accept suicide as the outcome of a decision making process most likely influenced by other processes such as socio- and environmental processes – processes by nature are dynamic; e.g. see Kral[22].

## References

1. De Leo, D. (2002) Why are we not getting any closer to preventing suicide? *Br J Psychiatry*. **181**, 372-4.
2. Institute of Medicine. US Report Calls for Improved Suicide Research. (2002) Washington: MEDLINEplus: http://www.nlm.nih.gov/medlineplus/news/fullstory_9705.html
3. Cutcliffe, JR. (2003) Research endeavours into suicide: a need to shift the emphasis. *Br J Nurs* **12(2)**, 92-99.
4. Blair-West, GW., Mellsop, GW., and Eyeson-Annan, ML. (1997)). Down-rating lifetime suicide risk in major depression. *Acta Psychiatr Scand* **95(3)**, 259-263.
5. Khan, A., Warner, HA., and Brown, WA. (2000) Symptom Reduction and Suicide Risk in Patients Treated With Placebo in Antidepressant Clinical Trials: An Analysis of the Food and Drug Administration Database. *Arch Gen Psychiatry* **57,** 311-317.
6. Hall, WD., Mant, A., Mitchell, PB., Rendle, VA., Hickie, I.B., and McManus, P. (2003) Association between antidepressant prescribing and suicide in Australia, 1991-2000: trend analysis. *BMJ*, 326.
7. Hakko, H., Rasanen, P., Tiihonen, J., and Nieminen, P. (2002) Use of statistical techniques in studies of suicide seasonality, 1970 to 1997. *Suicide Life Threat Behav.* **32(2),** 191-208.
8. Shahtahmasebi, S. "Suicides by Mentally Ill People" (2003) *TheScientificWorldJournal* **3**:684-693.
9. Maris, RW., Berman, AL., and Silverman, MM. (2000) Comprehensive textbook of suicidology. New York: Guilford Press.
10. Maris, RW., (1981) Pathways to suicide: A survey of self- destructive behaviours. Baltimore: Johns Hopkins Univ Press.
11. Wasserman, D. (2001) Suicide: an unnecessary death. London: Martin Dunitz.
12. Shahtahmasebi, S.and Berridge, D. Teenage smoking. (2005) A longitudinal analysis. *Int J Adolesc Med Health* **17(2)**.137-55.

13. Shahtahmasebi, S. (2004) Quality of life: A longitudinal analysis of correlates of morale in old age. *TheScientificWorldJournal* **4**, 100-110.
14. Brunner, J., Parhofer, K.G., Schwandt, P., Bronisch, T. (2002) Cholesterol, essential fatty acids, and suicide. *Pharmacopsychiatry* **35(1)**, 1-5.
15. Ainiyet, J. and Rybakowski, J. (1996) Low concentration level of total serum cholesterol as a risk factor for suicidal and aggressive behavior. *Psychiatria Polska*; **30(3),** 499-509
16. Draper, B.M. (1995) Prevention of suicide in old age. *Medical Journal of Australia* **162(10**, 533-534.
17. Beautrais, A.L. (2000) Risk factors for suicide and attempted suicide among young people. *Aust NZ J Psychiatry* **34(3),** 420-36.
18. Beautrais, A.L., Joyce, P.R., and Mulder, R.T. (1994) The Canterbury suicide project: Aims, overview and progress. *Community Mental Health in New Zealand* **8(2),** 32-39.
19. Beautrais, A.L. (2001) Suicides and serious suicide attempts: two populations or one? psychological medicine **31,** 837-845.
20. Beautrais, A.L. (1996) Serious suicide attempts in young people: a case control study [PhD]. Christchurch: Christchurch School of Medicine.
21. Ventegodt, S., Merrick, J., and Andersen, N.J. (2003) Quality of life as medicine: a pilot study of patients with chronic illness and pain. *TheScientificWorldJOURNAL* **3**, 520-532.
22. Kral, MJ. (1994) Suicide as social logic. Suicide & Life-Threatening Behavior **24(3)**, 245-55.

## BIOSKETCH

**Said Shahtahmasebi** is currently Principal Consultant, RaDiSol (R & D integrated Solutions) and Research Centre Director, The Good Life Research Centre Trust, Christchurch, New Zealand. His work experiences are mainly in the UK and cover a number of fields including public health, mental health, nursing, operational research, food and nutrition, gerontology. His area of interests and expertise are related to longitudinal modelling of health related behaviour. He has founded the Good Life Research Centre Trust to promote holistic research methodology to investigate health, social and behavioural issues relevant to the community (see http://b.1asphost.com/RaDiSol/). Email: said2@slingshot.co.nz
